# *In Vitro* Antioxidant, Anti-Diabetes, Anti-Dementia, and Inflammation Inhibitory Effect of *Trametes pubescens* Fruiting Body Extracts

**DOI:** 10.3390/molecules21050639

**Published:** 2016-05-16

**Authors:** Kyung Hoan Im, Trung Kien Nguyen, Jaehyuk Choi, Tae Soo Lee

**Affiliations:** Division of Life Sciences, Incheon National University, (Songdo-dong) 119 Academy-ro, Yeonsu-gu, Incheon 406-772, Korea; khim61@inu.ac.kr (K.H.I.); tknguyen@inu.ac.kr (T.K.N.); jaehyukc@inu.ac.kr (J.C.)

**Keywords:** anti-diabetes, anti-inflammation, antioxidant, anti-dementia, *Trametes pubescens*

## Abstract

*Trametes pubescens*, white rot fungus, has been used for folk medicine in Asian countries to treat ailments such as cancer and gastrointestinal diseases. This study was initiated to evaluate the *in vitro* antioxidant, anti-diabetes, anti-dementia, and anti-inflammatory activities of *T*. *pubescens* fruiting bodies. The 1,1-diphenyl-2-picryl-hydrazyl (DPPH) free radical scavenging activities of *T. pubescens* methanol (ME) and hot water (HWE) extracts (2.0 mg/mL) were comparable to butylated hydroxytoluene (BHT), the positive control. However, the chelating effects of ME and HWE were significantly higher than that of BHT. The HWE (6 mg/mL) also showed comparable reducing power to BHT. Eleven phenol compounds were detected by high performance liquid chromatography (HPLC) analysis. The α-amylase and α-glucosidase inhibitory activities of the ME and HWE of the mushroom were lower than Acarbose, the standard reference; however, the inhibitory effects of the mushroom extracts at 2.0 mg/mL were moderate. The acetylcholinesterase (AChE) and butyrylcholinesterase (BChE) inhibitory effects of ME and HWE were moderate and comparable with galanthamine, the standard drug to treat early stages of Alzheimer’s disease (AD). The ME had a neuroprotective effect against glutamate-induced PC-12 cell cytotoxicity at the concentration range of 2–40 μg/mL. The mushroom extracts also showed inflammation inhibitory activities such as production of nitric oxide (NO) and expression of inducible nitric oxide synthase (iNOS) in lipopolysaccharide (LPS)-induced murine macrophage-like cell lines (RAW 264.7) and significantly suppressed the carrageenan-induced rat paw-edema. Therefore, fruiting body extracts of *T*. *pubescens* demonstrated antioxidant related anti-diabetes, anti-dementia and anti-inflammatory activities.

## 1. Introduction

Mitochondria are the main organelles producing energy of the cell in the form of adenosine triphosphate (ATP) by oxidative phosphorylation (OXPHOS), and are also involved in signal transduction of cellular metabolic pathways. In addition to these activities, mitochondria play crucial roles for generating reactive oxygen species (ROS), the regulation of intracellular Ca^2+^, and programmed cell death (apoptosis). Although intracellular enzymes and other antioxidant defense system keep ROS concentrations under control, excessively produced ROS from mitochondria due to the incomplete processing of oxygen and free electrons released from electron transport chain (ETC) during OXPHOS were associated with chronic diseases such as diabetes, cardiovascular, ageing, inflammation, cancers, and neurodegenerative diseases [1]. Antioxidants are compounds which terminate the action of free radicals, thereby protecting the body from oxidative damages. Many researchers have demonstrated that vitamins and polyphenols from plant sources including fruits, vegetables, grains, roots, tea, coffee, and wine showed good antioxidant activities, and could protect mitochondria from dysfunction and apoptosis [2,3]. Recently, it was found that edible and wild mushrooms possess vitamins and polyphenol compounds and exhibit strong antioxidant activities [4,5]. Therefore, it is necessary to find new potent antioxidants from other mushroom sources.

Diabetes mellitus (DM) is a chronic metabolic disease caused by the insufficient production of insulin from β-cells of the pancreas or reduced sensitivity of cells to insulin. DM is characterized by high levels of glucose in the blood and causes the highest rates of mortality worldwide. The increased blood glucose levels leads to acute and chronic complications such as blindness, kidney failure, and hepatic and cardiovascular diseases. Recently, it was also found that type 2 diabetes was associated with the dysfunction of mitochondria. The alterations in mitochondrial oxidative activity may cause an imbalance between oxidative capacity and nutrient load, and the induction of insulin resistance and insufficient secretion of insulin [6]. To date, there is no satisfactory therapy available to cure type 2 DM. Several drugs, α-glucosidase inhibitors such as acarbose, voglibose, and miglitol, are now available to treat the patients who suffer from post-prandial hyperglycemia. These type of drugs inhibited degradation of carbohydrates in the digestive system, thereby reducing the glucose absorption by the cells and decreasing the blood glucose level. However, these drugs are associated with side effects such as yellow eyes or skin, and gastrointestinal disturbances including abdominal or stomach pains, diarrhea, passing of gas, thus searching for new natural anti-diabetic compounds is essential to overcome DM problems [7].

Alzheimer’s disease (AD) is the most common cause of dementia. Dementia refers to symptoms including memory loss and difficulties with thinking or language. These symptoms occur when certain diseases, including AD, damage the brain. The enzyme acetylcholinesterase (AChE) hydrolyzes acetylcholine (ACh), which is located at cholinergic brain synapses [8]. Recent evidence in scientific research has suggested that the dysfunction of mitochondria caused by oxidative stress were associated with AD and Parkinson’s disease (PD) [9]. An acetylcholinesterase inhibitor (AChE), anti-cholinesterase, is a chemical that inhibits acetylcholinesterase from breaking down acetylcholine, thereby increasing the action of acetylcholine. AChE inhibitors can increase cholinergic transmission by preventing the hydrolysis of Ach, thereby making more ACh available at the cholinergic synapse. This makes inhibitors of AChE the most potent means of treating cognitive symptoms of AD. Donepezil, rivastigmine, galantamine, physostigmine, and tacrine are the commercially available AChE inhibitors for treating early stages of AD. However, for clinical use, these drugs are associated with side effects such as loss of appetite, dizziness, headache, vomiting, and diarrhea [10]. As such, the search for potent natural products that selectively inhibit AChE activity without side effects are necessary.

Inflammation is considered as a part of the complex biological response to remove injury or harmful stimuli such as ROS, pathogens, damaged cells, or irritation by thermal heat, ultraviolet light or ionizing radiations. Macrophages are large specialized cells that engulf and digest cellular debris, microbes, and cancer cells using a process called phagocytosis. In response to stimuli, including lipopolysaccharide, macrophages play a critical role in the inflammatory response by producing a variety of factors, such as nitric oxide (NO), prostaglandin mediators, and pro-inflammatory cytokines (TNF-α, IL-1β, IL-6) [11]. Chronic inflammation can cause various degenerative disorders, including arthritis, cancer, dementia, diabetes and multiple sclerosis, and is also involved in the pathogenesis of mitochondrial dysfunction [12,13]. Recently, non-steroidal anti-inflammatory drugs (NSAIDs) are among the most widely prescribed medications in clinical practice worldwide. However, many studies have shown that the long-term administration of NSAIDs has the potential for significant side effects on the gastrointestinal tract causing numerous harmful effects such as mucosal lesions, bleeding, and peptic ulcers [14]. Thus, it is necessary to develop safe and effective anti-inflammatory agents from natural sources including mushrooms.

*Trametes pubescens*, a mushroom belonging to the family Polyporaceae of the order Polyporales in the Basidiomycota, is used in folk medicine in Asian countries [15]. In spite of the importance of this mushroom as a natural fork medicine, there have only been a few studies on its physiologically relevant activities [16,17]. Thus, this study was initiated to assess the antioxidant, anti-diabetes, anti-dementia, and inflammation inhibitory effects of *T. pubescens* fruiting body extracts. The constituent of phenolic compounds of the mushroom was also analyzed.

## 2. Results and Discussion

### 2.1. Antioxidant Activity Assay

#### 2.1.1. DPPH Radical Scavenging Activity

**Figure 1:**
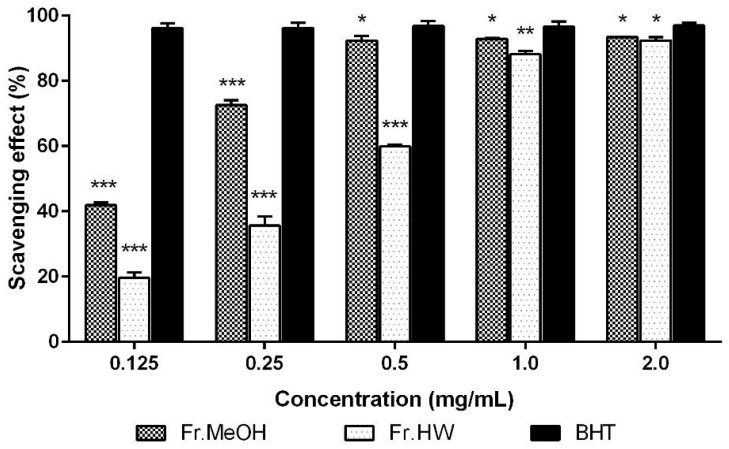
The 1,1-diphenyl-2-picryl-hydrazyl (DPPH) scavenging activities of methanol (ME) and hot water (HWE) extracts from fruiting bodies of *Trametes* *pubescens* on 1,1-diphenyl-2-picrylhydrazyl. Values are means ± S.D (*n* = 3). Fr.MeOH, fractions extracted with 80% methanol; Fr.HW, fractions extracted with hot water; BHT, butylated hydroxytoluene. *** *p* ≤ 0.001; ** *p* ≤ 0.01; * *p* < 0.05 *vs.* BHT.

The 1,1-diphenyl-2-picryl-hydrazyl (DPPH) free radical scavenging activities of the ME and HWE from *T. pubescens* fruiting bodies increased as concentration of the extracts increased. The radical scavenging activity of methanol (ME) and hot water (HWE) extracts at 0.125–2.0 mg/mL ranged from 41.91% to 93.45%, and from 9.62% to 92.38%, respectively. However, at the range of same concentration of the positive control, butylated hydroxytoluene (BHT) showed excellent scavenging ability (96.19%–96.97%) (Figure 1). In general, the DPPH scavenging activity of the ME was higher than that of HWE in the range of 0.5–1.0 mg/mL (*p* < 0.05). The highest scavenging activity (93.45%) was observed in the ME (2.0 mg/mL), however, this value was significantly lower than that of BHT. These results suggest that ME of the mushroom contained good radical scavenging effects, whereas the HWE had moderate scavenging activity at the concentrations tested.

Sumathy *et al.* [18] reported that the DPPH scavenging activities of methanol and chloroform extracts of *Pleurotus sajor-caju* fruiting bodies were 85%–94% and 83%–89% at 1.0–5.0 mg/mL, respectively. They further documented that the methanol and chloroform extracts of the carcophores of *P*. *citrinopileatus* had scavenging effects of 88%–96% and 86%–92%, respectively, at the concentrations tested. Mau *et al.* [19] found that the radical scavenging abilities of methanol extracts from *Grifola frondosa*, *Hericium erinaceus*, *Tricholoma giganteum*, and *Dictyophora indusiata* ranged from 63.3%–92.1% at a concentration of 6.4 mg/mL. Therefore, we conclude that the DPPH scavenging effects of the ME of *T. pubescens* fruiting body would be beneficial for human health.

#### 2.1.2. Metal Chelating Effects

**Figure 2:**
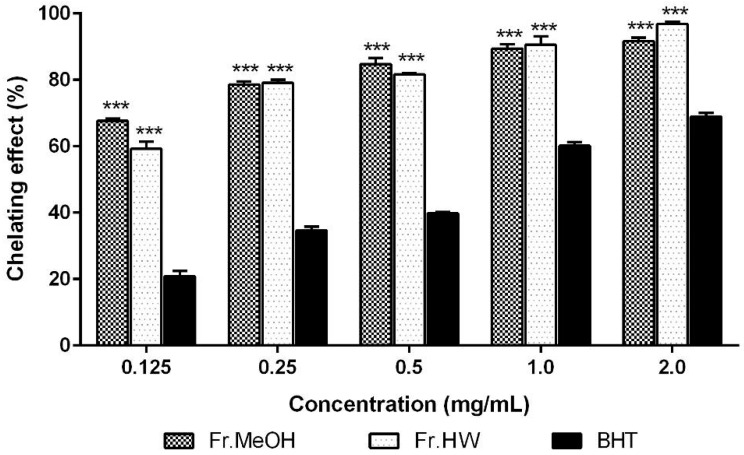
Chelating effect of methanol and hot water extract from fruiting bodies of *Trametes pubescens* at different concentrations. Values are means ± S.D (*n* = 3). Fr.MeOH, fractions extracted with 80% methanol; Fr.HW, fractions extracted with hot water; BHT, butylated hydroxytoluene. *** *p* ≤ 0.001 *vs.* BHT.

The metal chelating activity of the ME and HWE from *T. pubescens* fruiting bodies was investigated. The chelating effects of ME and HWE at five different concentrations (0.125–2.0 mg/mL) ranged from 67.66%–91.62%, and 59.23%–96.85%, respectively (Figure 2), demonstrating an increase in chelation in accord with increasing extract concentrations. The highest chelating activity (96.85%) was observed in the 2.0 mg/mL concentration of the HWE. At this concentration, slightly lower activity was detected in the ME (91.62%), whereas the chelating ability of BHT (68.87%) was significantly lower than those of both ME and HWE (*p* < 0.01).

Sarikurkcu *et al.* [20] reported that chelating activity on ferrous ion by the methanol extract of *Amanita caesarea*, *Clitocybe* *geotropa*, and *Leucoagaricus* *pudicus* were 74.1%, 37.2%, and 97.6% at 2.0 mg/mL, respectively. The chelating effects of methanol extracts from *Auricularia* *fuscosuccinea*, *Auricularia* *polytricha*, and *Tremella* *fusiformis* at 2.0 mg/mL were reported to be 91.28%, 88.04%, and 86.92%, respectively [21]. The ME and HWE determined herein exhibited significantly higher metal chelating activity than that of the standard reference, BHT, at the 0.125–2.0 mg/mL concentrations. Further, the ME and HWE of *T*. *pubescens* demonstrated higher chelating abilities than all mushrooms mentioned above except *L*. *pudicus*.

Ferrous ions are regarded as the strongest pro-oxidant among various metal ions. Chelating activity of ferrous ions can prohibit free radical generation and protect cells from oxidative damage by removing iron, which may be involved in free radical generation [22]. As ferrous ions are a potent pro-oxidant, the strong chelating activity found in *T*. *pubescens* fruiting bodies can be used as a natural antioxidant agent.

#### 2.1.3. Reducing Power

**Figure 3:**
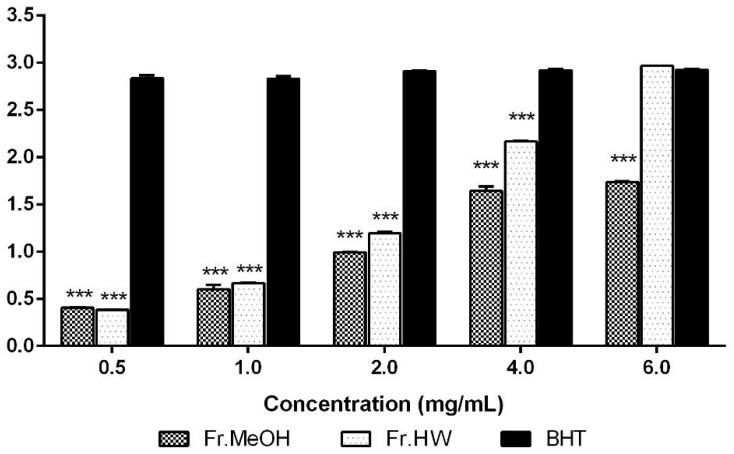
Reducing power of methanol and hot water extract from fruiting bodies of *Trametes pubescens* at different concentrations. Values are means ± S.D (*n* = 3). Fr.MeOH, fractions extracted with 80% methanol; Fr.HW, fractions extracted with hot water; BHT, butylated hydroxytoluene; *** *p* ≤ 0.001 *vs.* BHT.

The reducing powers of the ME and HWE from *T. pubescens* were analyzed and compared to BHT, the standard reference, at a range of 0.5–4.0 mg/mL. At these concentrations, the ME (0.40–1.64) and HWE (0.383–2.16) showed significantly lower reducing power compared with BHT (2.83–2.92) (*p* < 0.001); however, the reducing power of the HWE at 6.0 mg/mL concentration was almost the same as BHT (Figure 3). These results show that the reducing power of the both extracts from *T*. *pubescens* increased steadily as the extract concentrations increased, whereas the reducing power of BHT increased slowly as the concentration increased.

Lee *et al.* [23] documented that a ME and HWE from carcophores of *Pleurotus citrinopileatus* exhibited a reducing power of 1.03 and 1.10 at 5 mg/mL, respectively, which is significantly lower than the ME and HWE tested herein, 1.64 and 2.16 at 4.0 mg/mL, respectively. The reducing ability of the *Hypsizygus* *marmoreus* hot water extract was 0.99 at 5 mg/mL concentration [24], whereas those of *A*. *fuscosuccinea*, *A*. *polytricha*, and *T*. *fuciformis* were reported to be 2.19, 1.21, and 0.39 at 2.0 mg/mL, respectively [21].

Our results indicate that *T. pubescens* has lower reducing power than the above-mentioned mushrooms, except *P*. *citrinopileatus*, *H. marmoreus*, and *T. fuciformis*. Generally, the reducing power of mushrooms are associated with their hydrogen-donating ability [24]. Therefore, the *T*. *pubescens* fruiting body possesses good amounts of reducing properties, which might react with free radicals and thereby exert antioxidant activity.

### 2.2. HPLC Analysis of Phenolic Compounds

**Figure 4:**
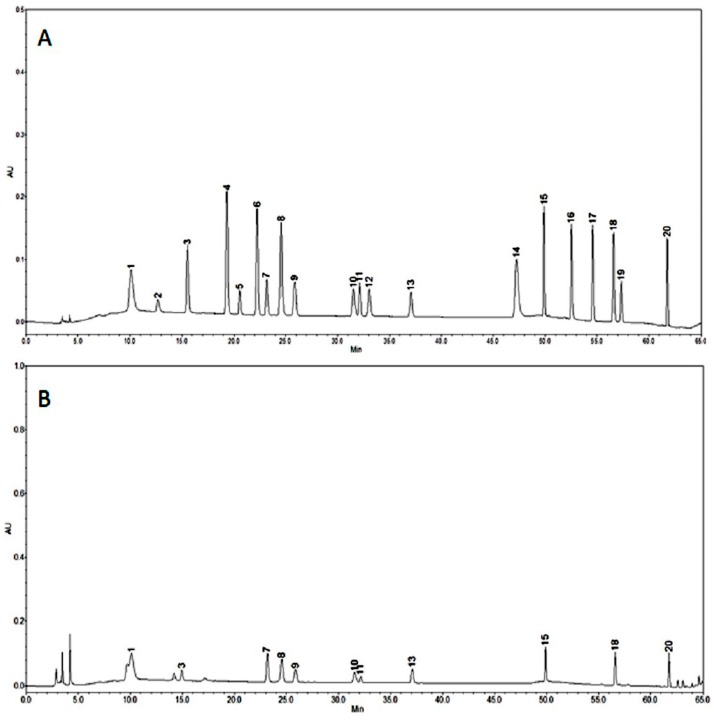
High performance liquid chromatography (HPLC) analysis of phenolic compounds. (**A**) standard compounds; (**B**) *Trametes* *pubescens*; 1, gallic acid; 2, homogentisic acid; 3, protocatechuic acid; 4, (+)-catechin; 5, chlorogenic acid; 6, (−)-epicatechin; 7, (−)-epigallocatechin gallate; 8, caffeic acid; 9, vanillin; 10, rutin hydrate; 11, *p*-coumaric acid; 12, ferullic acid; 13, naringin; 14, myricetin; 15, resveratrol; 16, quercetin; 17, naringenin; 18, kaempferol; 19, formonoentin; 20, biochanin-A.

High performance liquid chromatography (HPLC) was employed to determine the phenolic compound content of the extracts obtained from *T. pubescens* fruiting bodies. Eleven phenolic compounds were detected from the fruiting body of *T* *pubescens* with the total concentration of 86.61 μg/g (Figure 4). The phenolic compounds detected included gallic acid (18.8 μg/g), protocatechuic acid (2.92 μg/g), (−)-epigallocatechin gallate (14.8 μg/g), caffeic acid (4.81 μg/g), rutin hydrate (7.11 μg/g), *p*-coumaric acid (3.58 μg/g), naringin (11.09 μg/g), resveratrol (2.34 μg/g), kaempferol (6.74 μg/g), and biochanin-A (7.19 μg/g) (Figure 4B). The lowest and highest phenolic compound concentrations were protocatechuic acid and gallic acid, respectively. These results were comparable with those of previous findings in edible and medicinal mushrooms, where mushroom species had three to five different types of phenolic compounds, with gallic acid and protocatechuic acid being the most common [25].

Phenolic compounds are classified as simple phenols, phenolic acids, and polyphenols. Flavonoids are a group of polyphenolic compounds with well-known beneficial properties to health, such as free radical scavenging, hydrolytic inhibition, and anti-inflammatory activity [26,27]. Several papers have shown a positive correlation between phenolic compound concentrations and antioxidant activities. In general, phenolic compounds possess one or more aromatic rings with large numbers of hydroxyl groups, and thereby display a diverse structural diversities, exhibit radical scavenging, metal ion chelating, and reducing power, which resulted in good antioxidant effects [28,29].

### 2.3. In Vitro Anti-Diabetic Assay

#### 2.3.1. α-Amylase Inhibitory Activity

**Figure 5:**
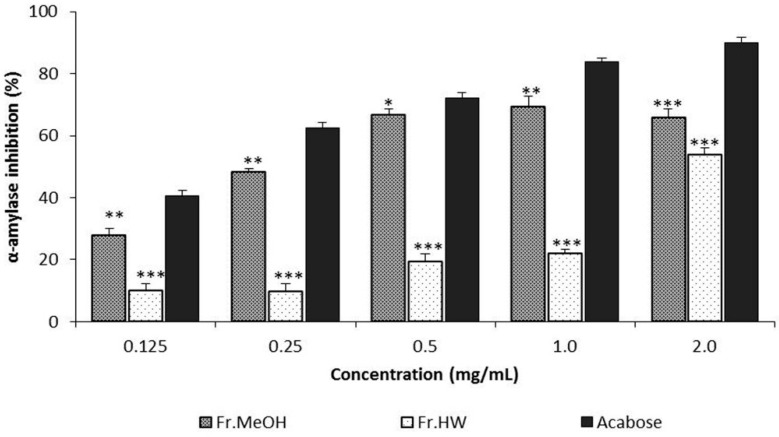
The α-amylase inhibitory activity of methanol and hot water extract of *Trametes pubescens* fruiting bodies. Values are means ± S.D (*n* = 3). Fr.MeOH, fractions extracted with 80% methanol; Fr.HW, fractions extracted with hot water; *** *p* ≤ 0.001, ** *p* ≤0.01, * *p* ≤ 0.05 *vs.* Acarbose.

The α-amylase inhibitory effects of the ME and HWE from *T. pubescens* fruiting bodies increased steadily with increasing concentration of *T. pubescens*. The inhibitory effects of the ME and HWE ranged from 27.75% to 69.46%, and from 10.05% to 53.82% at 0.125–2.0 mg/mL, respectively. However, Acarbose, the stand reference, showed excellent α-amylase inhibitory ability, ranging from 40.61% to 89.91% at 0.125–2.0 mg/mL (Figure 5). In general, the α-amylase inhibitory activity of the ME was significantly higher than that of HWE in the concentration range of 0.5–2.0 mg/mL (*p* < 0.05). The highest inhibition activity (69.46%) was observed in the ME at 2.0 mg/mL, however, this inhibitory activity was significantly lower than that of Acarbose, the reference drug. The results showed that the ME of mushroom had good inhibitory activity, whereas the HWE exhibited moderate activity at the concentrations examined. Santhoshkumar and Nagarajan [30] reported that α-amylase inhibitory effects of methanol extract of *Ganoderma lucidum* fruiting bodies were 21.92% to 94.46% at 0.2–1.0 mg/mL, respectively, whereas Pandimeena [31] documented that α-amylase inhibitory activity of methanolic extract of *Pleurotus florida* fruiting bodies were 13.78%–26.5% at the 0.25–1.0 mg/mL, respectively [31]. Our results demonstrated that ME of *T*. *pubescens* fruiting bodies possess relatively effective α-amylase inhibitory activities at 0.125–0.5 mg/mL concentrations compared with the mushrooms described above.

#### 2.3.2. α-Glucosidase Inhibitory Activity

**Figure 6:**
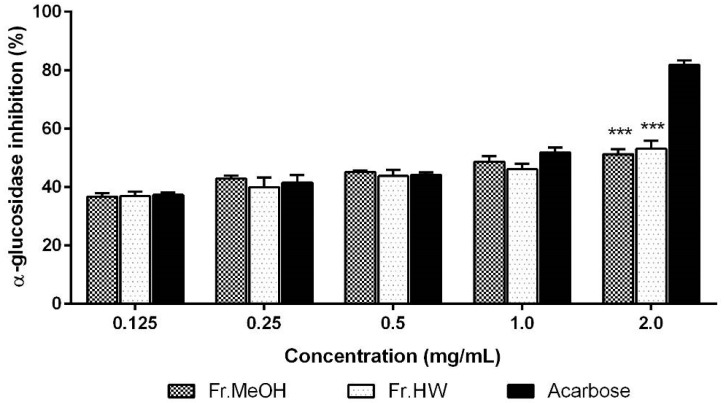
The α-glucosidase inhibitory activity of methanol and hot water extract from fruiting bodies of *Trametes pubescens*. Values are means ± S.D (*n* = 3). Fr.MeOH, fractions extracted with 80% methanol; Fr.HW, fractions extracted with hot water; *** *p* ≤ 0.001 *vs.* Acarbose.

The control of the early stage of DM is crucial for preventing chronic DM development. Several synthetic drugs are now available to treat DM, including α-glucosidase inhibitors (AGIs). AGIs work in the gastrointestinal tract by inhibiting digestion of starch, thereby increase the glycemic control and postprandial hyperglycemia modulation [32]. In this experiment, α-glucosidase inhibitory effects of fruiting body extracts of *T. pubescens* increased gradually with the increasing extract concentration. The inhibitory activities of ME and HWE ranged from 36.64% to 51.24%, and from 36.88% to 53.05% at 0.125–2.0 mg/mL, respectively. In contrast, Acarbose, the reference drug, exhibited the highest inhibitory activity at 0.125–2.0 mg/mL, ranging from 37.27% to 81.81% (Figure 6). The α-glucosidase inhibitory effects of both ME and HWE were similar in the range of 0.5–2.0 mg/mL concentration (*p* < 0.05). The highest inhibitory activity (53.05%) was detected in the HWE (2.0 mg/mL), however, this inhibitory activity was significantly lower than that of Acarbose. These results indicate that ME and HWE of the mushroom possessed moderate inhibitory activities against α-glucosidase. Santhoshkumar and Nagarajan [30] found that the inhibition of α-glucosidase by the methanol extract of *Ganoderma lucidum* fruiting bodies were 32.74%–86.53% at 0.2–1.0 mg/mL, which exhibited higher α-glucosidase inhibitory activity compared to our experimental results. Yin *et al.* [33] reported that constituents exhibiting α-glucosidase inhibitory activity from medicinal plants consist of various components, including terpenes, alkaloids, quinines, flavonoids, phenols, phenylpropanoids, sterides, and other types of compounds. Su *et al.* [34] found that major active components exhibiting α-glucosidase inhibition from fruiting bodies, including *Grifola frondosa*, *Hericium erinaceum*, *Agaricus* *blazei*, *Ganoderma lucidum*, *Coriolus versicolor*, and *Phellinus linteus*, were composed of oleic and linoleic acids. Therefore, it is suggested that the moderately high concentration of phenolic and other compounds present in the mushroom extracts may contribute to moderately higher α-glucosidase inhibitory activity.

### 2.4. Anti-Dementia Assay

#### 2.4.1. Anti-Acetylcholinesterase Activity

**Figure 7:**
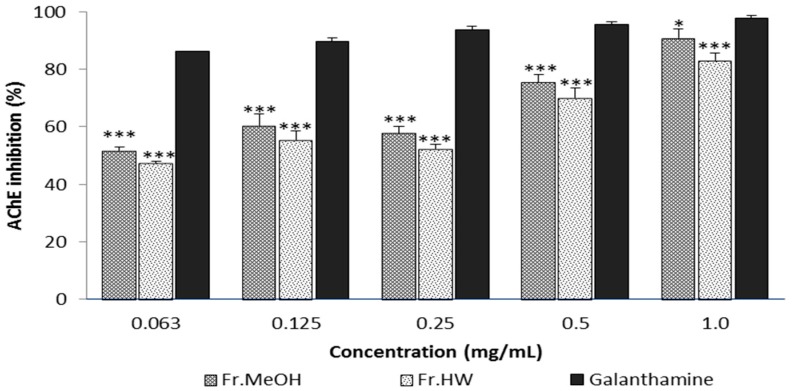
Acetylcholinesterase inhibitory activity of methanol and hot water extract of *T*. *pubescens* fruiting bodies. Values are means ± S.D (*n* = 4). Fr.MeOH, fractions extracted with 80% methanol; Fr.HW, fractions extracted with hot water. *** *p* ≤ 0.001; * *p* < 0.05 *vs.* galanthamine.

AChE, known as acetylhydrolase, catalyzes the hydrolysis of acetylcholine, one of the neurotransmitters found in the cholinergic brain synapses, where AChE serves to terminate synaptic transmission. The activities of AChE can lead to neurological disorders such as AD, senile dementia, and ataxia [35]. The anti-AChE effects from fruiting body extracts of *T*. *pubescens* were investigated herein. At concentrations ranging from 0.063 to 1.0 mg/mL, the inhibitory effects of the ME and HWE from the *T*. *pubescens* fruiting body ranged from 51.70% to 90.94%, and from 47.21% to 83.15%, respectively (Figure 7), and demonstrated concentration-dependent inhibition. The AChE inhibitory effect obtained from the ME was higher than that of the HWE at 0.063–1.0 mg/mL, whereas the inhibitory effects of galanthamine (97.80%), the standard reference, was significantly higher (*p* < 0.01) than both extracts.

Previous reports documented that phenolic acids and flavonoid derivatives such as gallic acid, chlorogenic acid, quercetin, caffeic acid, ferulic acid, ellagic acid, catechin, rutin, and luteoin-7-rutinoside, are strong inhibitors of the AChE enzyme [36]. Our experimental results (Figure 4B) also indicate that the fruiting body of *T. pubescens* contains 11 different phenolic compounds including gallic acid (18.8 μg/g), caffeic acid (4.81 μg/g), and rutin hydrate (7.11 μg/g), which have strong AChE inhibitory potential. These results support the previous findings concerning phenolic acids and flavonoids mentioned above. Therefore, we conclude that the high AChE inhibitory activity found in the ME and HWE may be due to the polyphenolic compounds in the fruiting bodies.

#### 2.4.2. Anti-Butyrylcholinesterase Activity

**Figure 8:**
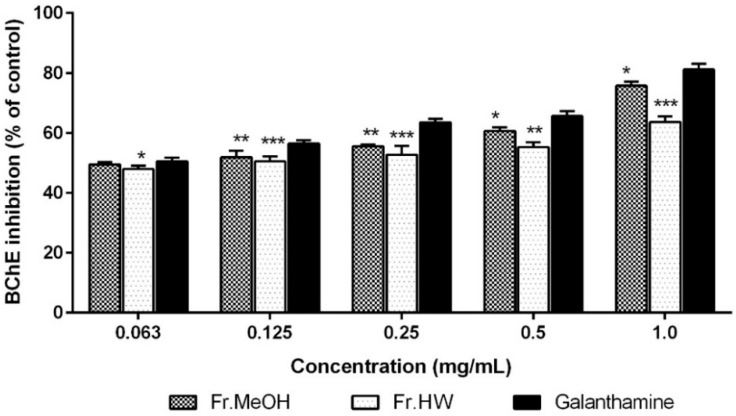
Butylrylcholinesterase (BChE) inhibitory activity of methanol and hot water extract of *T*. *pubescens* fruiting bodies. Values are means ± S.D (*n* = 4). Fr.MeOH, fractions extracted with 80% methanol; Fr.HW, fractions extracted with hot water. *** *p* ≤ 0.001; ** *p* ≤ 0.01; * *p* < 0.05 *vs.* galanthamine.

The butyrylcholinesterase (BChE) inhibitory activities of ME and HWE from *T. pubescens* fruiting bodies were analyzed and compared with galanthamine, the standard reference. The BChE inhibitory effects of the ME (49.49%–75.73%) and HWE (47.93%–63.58%) were significantly lower than that of galanthamine (50.56%–81.12%) at 0.063–1.0 mg/mL (*p* < 0.001). The AChE inhibitory activity of both extracts increased with as their respective concentrations increased (Figure 8).

Orhan *et al.* [37] screened four phenolic acids and 10 flavonoid derivatives for BChE inhibitory activities and found chlorogenic acid, gallic acid, quercetin, genistein, leteolin-7-*O*-galactoside, naringin, silibinin, and silymarin to have inhibitory activities. In our experiment, two of those phenolic compounds, gallic acid and naringin, were found in the fruiting bodies of *T*. *pubescens*. These results could explain the high and moderate inhibitory activities of the fruiting body extracts of *T. pubescens* toward the BChE enzyme. 

#### 2.4.3. Glutamate-Induced Cytotoxicity

Glutamate is one of neurotransmitter in the central nervous system of mammals. However, excessively released glutamate in the system can lead to neuronal damage and cell death in a process known as excitotoxicity. A recent study suggested that the oxidative stress could cause mitochondrial dysfunction, and this event was mediated by glutamate-induced excitotoxicity and associated with neurodegenerative disorders [38]. 

**Figure 9:**
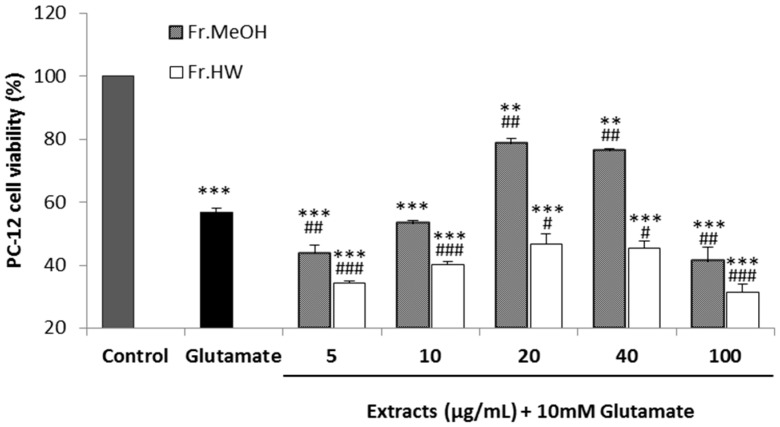
Glutamate-induced cytotoxicity activity of methanol and hot water extract from fruiting body of *Tramates pubescens* against pheochromocytoma cell line (PC-12). Values are means ± S.D (*n* = 4). *** *p* ≤ 0.001; ** *p* ≤ 0.01 *vs.* control group; ^###^ *p* ≤ 0.001; ^##^ *p* ≤ 0.01; ^#^ *p* ≤ 0.05 *vs.* glutamate.

A pheochromocytoma cell line (PC-12) derived from rat adrenal medulla, was used to evaluate the glutamate-induced cytotoxicity. The cell viability of PC-12 cells supplemented with 10 mM glutamate was 56.65% compared with the control, whereas the viabilities after 5, 10, 20, 40, and 100 μg/mL of ME supplementation were 43.63%, 53.32%, 78.73%, 76%, 35%, and 41.27%, respectively. In contrast, the viabilities of glutamate-treated PC-12 cells were 34.40%, 40.15%, 46.85%, 45.40%, and 31.47%, respectively, after treatment with the same concentrations of the HWE (Figure 9). These results suggest that the cytotoxicity of PC-12 cells induced by glutamate treatment was attenuated by the addition of the ME at the concentrations of 20 and 40 μg/mL, whereas the supplementation of 5, 10, and 100 μg/mL could not mitigate the cytotoxicity. However, the hot water extracts could not improve the cell viabilities compared with the glutamate-treated group at any concentration. The ME of *T. pubescens* only significantly alleviated the glutamate-induced cytotoxicity on PC-12 cells at 20 and 40 μg/mL.

Lee *et al.* [39] reported that PC-12 cell treatment with (−)-epigallocatechin gallate (EGCG), a polyphenol from green tea, reduced the glutamate-induced Ca^2+^ influx increase, while also increasing the viability of PC-12 cells against glutamate-induced cytotoxicity. Tan *et al.* [40] also found that various concentrations of biochanin-A from *Trifolium pratense* attenuated the glutamate-induced PC-12 cell cytotoxicity. In the present study, the ME extract significantly increased viability of PC-12 cells from glutamate-induced cytotoxicity, indicating that various phenolic compounds present in the mushroom fruiting body exerted free radical scavenging activities on the ROS generated by glutamate supplementation, and thereby protected the cells from death.

### 2.5. Inflammation Inhibitory Assay

#### 2.5.1. Production of NO

**Figure 10:**
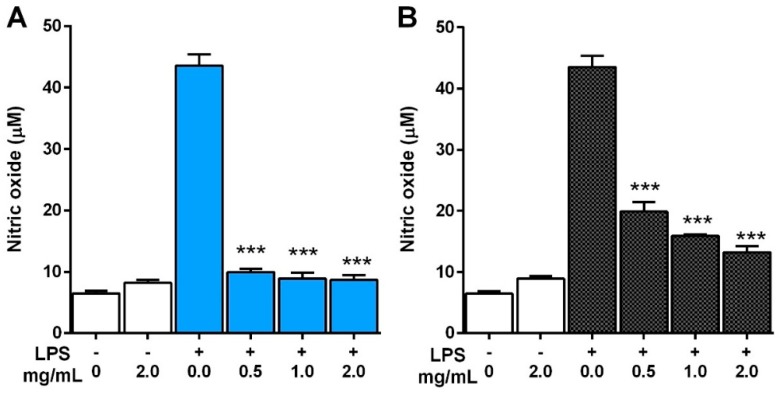
Inhibitory effect of *Trametes pubescens* fruiting bodies on lipopolysaccharide (LPS)-induced nitric oxide production in murine macrophage-like cell-lines (RAW 264.7): (**A**) methanol extract; (**B**) hot water extract. Nitric oxide in the culture medium was determined by the Griess reaction assay. Values are means ± S.D (*n* = 3). *** *p* < 0.001 *vs.* LPS-treated group.

Macrophages play pivotal roles in inflammatory process by producing several pro-inflammatory molecules, including NO. Evidence has indicated that excessively produced NO has been associated with various inflammation-related ailments such as arteriosclerosis, hypertension, and septic shock [41]. To evaluate the inhibitory effect of the mushroom extracts on the production of NO, lipopolysaccharide (LPS)-induced macrophage-like cell-lines (RAW 264.7) were used. The concentration of NO after treating LPS on RAW 264.7 macrophages for 24 h increased ~6.73-fold (6.47–43.55 μM), while the NO concentration from the varying *T*. *pubescens* extract concentration treatments decreased significantly in a concentration-dose dependent manner from 0.5 to 2 mg/mL (Figure 10).

RAW 264.7 macrophages treated only with 2 mg/mL of ME produced 8.21 μM of NO, which is comparable with the 6.47 μM of LPS non-treating group. However, LPS-treated RAW 264.7 macrophages supplemented with 2 mg/mL ME produced 8.67 μM of NO, indicating a 1.34-fold higher concentration than the control (Figure 10A). The RAW 264.7 macrophages treated with 2 mg/mL of HWE also showed a significant decrease in the production of NO by 69.58% compared with the group treated only LPS (Figure 10B). These results show that the inhibitory activity of the ME on the LPS-induced NO production in RAW 264.7 macrophages was significantly higher than that of the HWE. The higher inhibitory effect of NO production in ME over HWE may be due to some ingredient of the fruiting body exhibiting anti-inflammatory effects that were inactivated by hot water heating. The cytotoxic effect on RAW 264.7 macrophages was not observed from both of the extracts by the MTT test (data not shown). These experimental results suggest that the inhibition of NO production by treatment of the ME and HWE on RAW 264.7 macrophages was due to reduced expression of inducible nitric oxide (iNOS) protein. Song *et al.* [42] found that NO production in LPS-induced RAW 264.7 macrophages was suppressed by ethanolic extract of *Ganoderma lucidum* fruiting bodies in a concentration-dependent manner. Furthermore, Moro *et al.* [11] reported that the methanol extracts with rich phenolic compounds from fruiting bodies of wild edible mushrooms such as *Agaricus bisporus*, *Cantherellus cibarius*, *Lactarius deliciosus*, and *Craterellu*s *cornucopioides*, suppressed NO production in LPS-induced RAW 264.7 macrophages. Park *et al.* [43] documented that methanol extract of *Inonotus obliquus* attenuated NO production significantly on LPS-stimulated macrophages by inhibiting proteins and mRNA expressions of inducible nitric oxide (iNOS) and cyclooxygenase-2 (COX-2). It was reported that the strong anti-inflammatory components detected in mushrooms such as *P*. *ostreatus*, *Macrolepiota procera*, *Boletus impolitus*, and *Agaricus bisporus* were phenolic compounds including cinnamic acid, *p*-coumaric acid, and *p*-hydroxybenzoic acids and their derivatives [44]. Therefore, we conclude that the higher inhibitory effect on LPS-induced NO production from RAW 264.7 cells by *T*. *pubescens* fruiting body extracts was due to phenols and other compounds present in the mushroom extracts, and that the inhibitory effects herein were similar to those of mushrooms mentioned above.

#### 2.5.2. Western Blot Analysis

LPS-induced RAW264.7 macrophages showed a dose-dependent reduction in NO production after treatment with the ME and HWE of *T. pubescens*. This line of experiments was performed under the assumption that the suppressed NO production was due to suppression of iNOS expression. To investigate the expression of iNOS protein by the mushroom extracts, a Western blot analysis was conducted. Since the ME showed the highest inhibitory effect on the NO production in this experiment, LPS-stimulated RAW 264.7 macrophages treated with the ME was used for the Western blot analysis.

**Figure 11:**
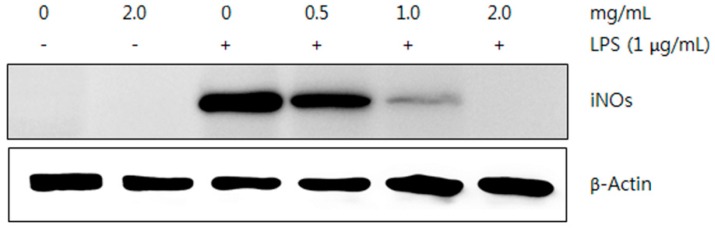
Inhibitory effect of methanol extract from *Trametes pubescens* fruiting bodies on LPS-induced expression of nitric oxide synthase (iNOS) in RAW 264.7 cells. β-Actin was used as an internal control.

The amount of iNOS protein decreased by treating ME in a dose-dependent manner, while the expression of β-actin protein was not changed, indicating that only iNOS expression was suppressed by the ME (Figure 11). Chen *et al.* [45] reported that the glycoprotein isolated from fruiting bodies of *Pleurotus citrinopileatus* inhibited the NO production and expression of iNOS in LPS-induced RAW 264.7 cell lines in a dose-dependent manner. Wu *et al.* [46] reported that the ethanol extract from *Armillariella mellea* fruiting bodies also suppressed NO production and expression of the iNOS protein in LPS-stimulated RAW 264.7 macrophages. These results suggest that reduced NO production and expression of the iNOS protein in the RAW 264.7 cells may be due to the mushroom extract treatment.

#### 2.5.3. Carrageenan-Induced Paw Edema

The process of carrageenan-induced edema can be classified by two phases. The initial phase is characterized by releasing histamine, serotonin, and kinin, while the late phase is involved in the release of bradykinin and prostaglandin. The release of histamine in the initial stage leads to the outward movement of proteins and fluid into the extracellular spaces, while the prostaglandin produced during the late phase is responsible for the edema formation and most of drugs for anti-inflammatory treatment are more effective in the late stage of edema development [47].

**Figure 12:**
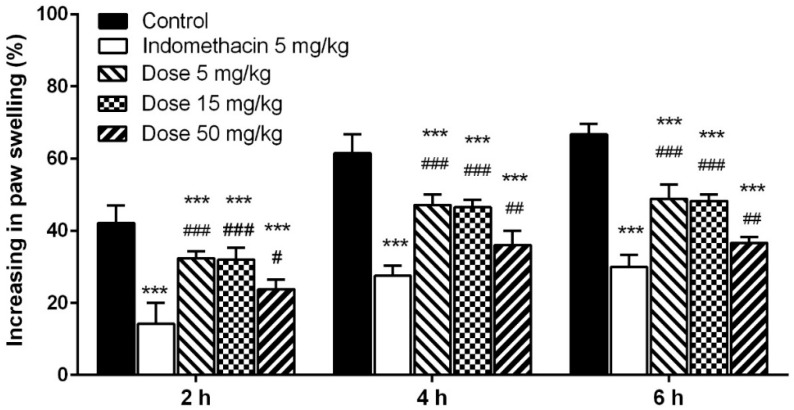
Effect of methanol extract from *Trametes pubescens* fruiting bodies on carrageenan-induced hind paw edema. Values are means ± S.D (*n* = 5). ^###^ *p* ≤ 0.001; ^##^ *p* ≤ 0.01; ^#^ *p* < 0.05 *vs.* indomethacin group. *** *p* ≤ 0.001 *vs.* control group.

The ME of *T*. *pubescens* exhibited concentration-dependent anti-inflammatory activity and significantly decreased the edema volume in the hind paw of rats (*p* < 0.001). As shown in Figure 12, 6 h after subcutaneous injection, indomethacin, the reference drug, reduced the rat paw edema significantly (55.09%), whereas the administration of ME (5, 15, and 50 mg/kg) caused a reduction of paw edema by 26.74%, *27*.67% and 46.05%, respectively. The 5–50 mg/kg doses of ME showed a highly significant reduction of the paw edema at 2, 4, and 6 h after the initiation of edema compared with the control (*p* < 0.001). Although the inhibitory effect of indomethacin on the carrageenan-induced edema of rats was significantly higher than that of the ME at a dose of 50 mg/kg (*p* < 0.01), the effects of ME were comparable. It was also found that methanol extract from *I*. *obliquus* significantly reduced volume of carrageenan-induced paw edema of rats compared with the control, however the inhibitory effect of the methanol extract was significantly lower than that of ibuprofen, the positive control [43].

Therefore, *in vitro* antioxidant and *in vivo* inflammation inhibitory effects found in *T*. *pubescens* fruiting bodies may be useful for treating inflammation related disorders.

## 3. Materials and Methods

### 3.1. Chemicals and Reagents

All phenolic standards, methanol, butylated hydroxytoluene (BHT), 1,1-diphenyl-2-picryl-hydrazyl (DPPH), ferric chloride, ferrous chloride, ferrozine, potassium ferricyanide, trichloroacetic acid, Folin–Ciocalteu reagent, 3,4-dihydroxy-l-phenylalanine, dimethyl sulfoxide, tris-HCL, tris base, glacial acetic acid, trichloroacetic acid, acarbose, *p*-nitrophenyl-α-d-glucopyranoside, 5,5′-dithiobis-(2-nitrobenzoic acid) (DTNB), galanthamine, lipopolysaccharides (LPS), and carrageenan were purchased from Sigma-Aldrich (St. Louis, MO, USA). The iNOS antibody was purchased from Santa Cruz Biotechnology Co, and an ECL kit was purchased from Amersham Co (GE Healthcare Life Sciences, Buckinghamshire, UK).All chemicals and solvents used for the experiment and high performance liquid chromatography (HPLC) were of analytical grade.

### 3.2. Experimental Animals

Inbred male rats (Sprague Dawley, five weeks old, 140–160 g) were obtained from Animal Inc., Seoul, Korea, and used for this experiment. Animals were housed under temperature at 23 ± 2 °C, relative humidity (50%–60%), and a 12/12 h dark and light cycle. A standard diet and water were supplied to the animals ad libitum. Animals were kept in an animal house one week for acclimation before experiment. Experimental procedures have been performed in accordance with the animal care guidelines of Animal Ethics Committee at the Incheon National University and the protocol was evaluated and approved by the committee (Ethics no. 117-2.28.2014).

### 3.3. Mushroom Extract

*T. pubescens* fruiting bodies were collected from Incheon City Grand Park and identified by Dr. Kyung-Rim Lee (mycologist). The voucher specimen (IUM 4605) was deposited in the “Culture Collection of Mushrooms”, Incheon National University. The *T*. *pubescens* fruiting bodies were fully air dried at 45 °C and ground into a fine powder.

To obtain the methanol extract (ME), 10 grams of the powder was extracted with 200 mL of 80% methanol at orbital shaker (150 rpm) for 24 h at 25 °C. Then, the mixture was filtered through filter paper. To obtain the hot water extract (HWE), 10 g of the powder was boiled for 3 h in 200 mL of distilled water, cooled to 25 °C and filtered with filter paper. Then, the remaining residue was extracted with 200 mL of methanol or distilled water twice as described above, respectively. The ME and HWE were evaporated to dryness at 40 °C under reduced pressure in a rotary evaporator and remaining solvent were removed with a freeze-drier.

### 3.4. Antioxidant Activity

#### 3.4.1. DPPH Radical Scavenging

DPPH free radical scavenging activity was measured according to the methods described by Galvez *et al.* [48] with minor modifications. One mL of 0.1 mM DPPH radical solution in methanol was mixed with 1 mL aliquots of varying mushroom extract concentrations (0.125, 0.25, 0.5, 1.0 and 2.0 mg/mL). The reaction was carried out by shaking vigorously and then allowing it to stand for 30 min in the dark before measuring the absorbance at 517 nm by a UV-Vis spectrophotometer. The percent inhibition of DPPH radicals was calculated as following formula:

Inhibition % = [(A_c_ − A_s_/A_c_)] × 100 (1)

where A_c_ is the absorbance of the control and A_s_ is the absorbance of the test compound. BHT was used as a standard reference.

#### 3.4.2. Chelating Effects on Ferrous Ions

The chelating ability of ferrous ions was investigated according to the method described by Sowndhararajan and Kang [49] with slight modification. Two mL of methanol aliquots of varying concentrations of the extracts (0.063, 0.125, 0.25, 0.5, and 1.0 mg/mL) were mixed with 0.05 mL of 2 mM FeCl_2_ solution. Then, 2.95 mL of methanol was added to adjust the total volume of 5 mL and allowed to stand for 10 min at 25 °C. Half a mL of varying extract concentrations were mixed with 0.5 mL of FeSO_4_ (0.12 mM) and 0.5 mL of ferrozine (0.6 mM). The reaction was carried out by shaking the mixture vigorously. After 10 min of incubation at 25 °C, the absorbance was measured by a UV-Vis spectrophotometer at 562 nm. The percent of the sample’s ability to chelate ferrous ions was calculated by following formula:

Ferrous ion chelating ability (%) = [(A_c_ − A_s_)/A_c_] × 100 (2)

where A_c_ is the absorbance of the control and A_s_ is the absorbance of the test compound. BHT was used as a standard reference

#### 3.4.3. Reducing Power Assay

The reducing power was investigated by the method of Glucin *et al.* [50]. Varying concentrations (0.5, 1.0, 2.0, 4.0, 6.0 mg/mL) of ME and HWE in methanol were mixed with 2.5 mL of 200 mM, sodium phosphate buffer (pH 6.6) and 2.5 mL of potassium ferricyanide (1%). The mixture was kept at 50 °C for 20 min. Then, the reaction mixture was acidified with 2.5 mL of trichloroacetic acid (10%), centrifuged at 650 rpm for 10 min and 5 mL of supernatant was mixed with deionized water (5 mL). Then, 1 mL of ferric chloride (0.1%) was added to the solution and the absorbance was measured at 700 nm in a spectrometer. BHT was used as standard reference.

### 3.5. Phenolic Compounds Analysis by HPLC

Twenty phenolic standard compounds such as chlorogenic acid, gallic acid, (+)-catechin, homogentisic acid, protocatechuic acid, (−)-epicathecin, (−)-epigallocathecin gallate, caffeic acid, vanillin, rutin hydrate, *p-*coumaric acid, ferulic acid, myricetin, naringin, resveratrol, quercetin, naringenin, kaempferol, fomononetin, and biochanin-A were obtained from Sigma-Aldrich (St. Louis, MO, USA). We prepared mushroom extract for phenolic compound analysis according to the method of Im *et al.* [51]. Alliance^®^ HPLC system 2695 (Waters, Milford, MA, USA) was used for the analysis of phenolic compound constituents of the mushroom extract. Reverse phase C_18_ column (XSELECT CSH™, 150 mm × 4.6 mm × 3.5 μm) was used for separation at 40 °C. The injection volume was 20 μL and the mobile phase consisted of solvent A (0.85% phosphoric acid) and solvent B (acetonitrile). The flow rate was 0.5 mL/min. The gradient employed solvent A (100%), then was adjusted to solvent A (93%) and solvent B (7%) for 5 min, A (91%)/B (9%) for 10 min, A (85%)/ B (15%) for 15 min, A (78%)/B (22%) for 30 min, A (75%)/B (25%) for 40 min, A (62%)/B (38%) for 45 min, and solvent B (100%) for 60 min. The running time was 65 min. The identification of phenolic compounds present in the extracts were determined by photodiode array detector (Waters 2988) at 280 nm by comparing retention times to those of the authentic standard references.

### 3.6. Anti-Diabetic Assay

#### 3.6.1. α-Amylase Inhibition

The α-amylase inhibitory activity was performed by the method as described by Tadera *et al.* [52] with minor modification. Two hundred μL of varying concentration of extracts (0.125–2.0 mg/mL) were prepared in 20 mM, pH 6.9 phosphate buffer and then mixed with 200 μL of porcine pancreatic α-amylase (0.5 mg/mL) and incubated at 25 °C for 10 min, and then 200 μL of starch solution (1%) was added and kept at 25 °C for 30 min. The reaction was stopped by adding 1.0 mL of dinitrosalicylic acid reagent (1.0 g of 3.5-dinitrosalicylic acid in 20 mL of 2 M NaOH + 50 mL distilled water + 30 g potassium sodium tartrate tetrahydrate). Then, the mixture was dissolved in distilled water to make a total volume of 100 mL, and incubated in a water bath (100 °C) for 5 min and cooled to room temperature. The reaction mixture was measured at 540 nm with a UV-Vis spectrophotometer. The α-amylase inhibitory activity was calculated using the following formula:

Percent Inhibition = [(A_c_ − A_s_)/A_c_] × 100 (3)

where A_c_ is the absorbance of the control reaction (containing all reagents except the test compound) and A_s_ is the absorbance of the test compound. Acarbose was used for the standard reference.

#### 3.6.2. α-Glucosidase Inhibition

One-hundred μL aliquots of varying extract concentrations (0.125, 0.25, 0.5, 1.0 and 2.0 mg/mL) were prepared in 100 mM phosphate buffer (pH 6.9) and 100 μL of 1.0 U/mL α-glucosidase enzyme solution were mixed and kept at 37 °C for 10 min. Then, 100 μL of 5 mM *p*-nitrophenyl-α-d-glucopyranoside solution was added and the reaction mixture was incubated at 37 °C for 10 min. Then, 20 μL of the mixture was diluted into 1.0 mL of deionized distilled water. The absorbance was measured at 405 nm by a UV-Vis spectrophotometer [52]. The α-glucosidase inhibitory activity was calculated using the formula (3).

### 3.7. Anti-Dementia Assay

#### 3.7.1. Anti-Acetycholinesterase Activity

Acetylcholinesterase inhibitory activity was determined by a slight modification of the method described by Orhan *et al.* [53]. Electric eel AChE (Type-VI-S, Sigma, C3389-500UN) was used as the enzyme source, while acetylthiocholine iodide was employed as the substrate for the reaction. The acetylcholinesterase activity was measured using 5,5-dithio-bis(2-nitrobenzoic) acid (DTNB). In brief, 120 μL of 100 mM sodium phosphate buffer (pH 8.0), 30 μL of sample solution dissolved in methanol at various mushroom concentration of extracts (0.063–1.0 mg/mL) and 30 μL of AChE (3 U/mL) were added and incubated at 25 °C for 30 min, and then 10 μL of DTNB (0.5 mM) was supplemented. The reaction was started by adding 10 μL acetylthiocholine iodide (0.71 mM). The hydrolysis of acetylthiocholine iodide was monitored at 412 nm in a 96-well microplate reader (SpectraMax 340PC, Sunnyvale, CA, USA). The percentage of AChE inhibition was calculated by comparing the sample reaction rates respective to the control (without sample) by the formula:

% inhibition activity of acetylcholinesterase = [(A_c_ − A_s_)/A_c_] × 100 (4)

where A_c_ is the enzyme activity without a test sample and A_s_ is the enzyme activity with a test sample). Galanthamine was used for the standard reference.

#### 3.7.2. Anti-Butyrylcholinesterase Activity

The inhibition effect of butyrylcholinesterase was determined by the method of Orhan *et al.* [53] with minor modification. Horse serum BChE (Sigma, C1057-1KU) was used as the enzyme source, while butyrylcholine iodide was employed as the substrate. DTNB was used to measure activity of butyrylcholinesterase. In brief, 120 μL of 100 mM sodium phosphate buffer (pH 8.0), 30 μL of sample solution dissolved in methanol with various mushroom extract concentrations (0.063–1.0 mg/mL) and 30 μL of BChE (0.35 U/mL) were added and incubated at 25 °C for 30 min, then 10 μL of 0.5 mM DTNB was supplemented. Then, the reaction was started by adding 10 μL of butyrylcholine iodide (0.2 mM). The hydrolysis of butyrylcholine iodide was monitored at 412 nm in a 96-well microplate reader (SpectraMax 340PC). Percent inhibition of BChE was calculated by comparing the reaction rates of samples relative to the control (without sample) using the formula:

% butyrylcholinesterase inhibitory activity = [(A_c_ − A_s_)/A_c_] × 100 (5)

where A_c_ is the enzyme activity without a test sample and A_s_ is the enzyme activity with a test sample. Galanthamine was used for the standard reference.

#### 3.7.3. Glutamate-Induced Cytotoxicity

Cytotoxicity induced by glutamate on PC-12 cell line was determined by modifying the method of Ma *et al.* [54]. PC-12 cells were seeded in a 96-well microplate at a density of 1 × 10^5^ cells/well. The cell line was cultured for 24 h, and the medium was then changed to contain 10 mM glutamate. After 12 h of incubation, the cells were treated with varying mushroom extract concentrations (5, 10, 20, 40, and 100 μg/mL) and cultured for 24 h. After addition of 10 μL of 3-(4,5-dimethylthiazol-2-yl)-2,5-diphenyltetrazolium bromide (MTT; 5 mg/mL) to each well, the cells were incubated for an additional 4 h and the supernatant removed. The resulting purple formazan crystals were dissolved in 200 μL of dimethyl sulfoxide (DMSO) and the absorbance was measured at 570 nm in a microplate reader.

### 3.8. Anti-Inflammatory Activities

#### 3.8.1. Inhibitory Effect on NO Production

The inhibitory effect of the ME and HWE on the production of NO was determined by the method of Choi *et al.* [55]. RAW 264.7 macrophage cells were plated into 96-well plates (5 × 10^5^ cells/well) and incubated for overnight at 37 °C with 5% atmospheric CO_2_. Then, the medium was replaced by fresh medium (0.2 mL) and incubated for 60 min. Then, LPS (1 μg/mL) was added to the culture medium and incubated for 24 h with or without various mushroom extract concentrations (0.5, 1.0, and 2.0 μg/mL). Fifty μL of supernatant was collected and used in the NO analysis. 

The accumulation of NO in the medium by RAW 264.7 cells was investigated by the Griess assay [35]. Briefly, 50 μg of cell culture medium was mixed with 50 μg of Griess reagent (equal volume of 1% sulfanilamide in 0.1% naphthylethylenediamide-HCl and 5% phosphoric acid (*v*/*v*)). The reaction mixture was left to incubate at 25 °C for 30 min. The absorbance was measured by a microplate reader at 540 nm, and the NO concentration was determined by sodium nitrite solution as standard concentration.

#### 3.8.2. Western Blot Analysis

The Western blot analysis of the ME was performed by the method described by Coruzzi *et al.* [56]. RAW 264.7 macrophage cells (5 × 10^5^ cells/well) were seeded in 98-well culture plates and treated with LPS alone or LPS (1 μg/mL) with various mushroom extract concentrations (0, 0.5, 1.0, and 2.0 mg/mL) and incubated for 24 h. Then, the cells were washed twice with ice-cold phosphate buffered saline (pH 8.0) and lysed in 250 μL of ice-cold buffer containing 20 mM Tris-HCl (pH 7.5), 1% Triton X-100, 137 mM NaCl, 2 mM ethylenediaminetetraacetic acid (EDTA), 1 mM sodium orthovanadate, 2 mM sodium pyrophosphate, 1 mM phenylmethylsulfonyl fluoride, 1 μg/mL leupeptin, 62.5 mM Tris (pH 6.8), 2% sodium dodecyl sulphate (SDS), 10% glycerol, 5% 2-mercaptoethanol, and protease inhibitors. The cell lysates were centrifuged at 10,000× *g* for 10 min at 4 °C. Total cellular proteins from supernatants were separated by 8% SDS-polyacrylamide gel electrophoresis and transferred onto a polyvinylidene difluoride (PVDF) membrane in 25 mM Tris, 20% methanol, and 192 mM glycine. The membranes were blocked with non-fat milk (5%) dissolved in tris-buffered saline (TBS) containing 0.1% Tween-20 (TTBS) at 4 °C for 1 h. After blocking, membranes were incubated overnight at 4 °C and then incubated at 25 °C with 0.5 g/mL anti-mouse iNOS antibodies (BD Biosciences, San Diego, CA, USA) for 2 h. The blotted membranes were washed and incubated with peroxidase-conjugated goat anti-mouse immunoglobulin G (IgG) in TTBS containing 5% non-fat milk for 1 h. Then, the blots were washed in TTBS three times and the iNOS protein expression was detected by the Amersham ECL Prime Western blotting detection system (GE Healthcare Life Sciences, Buckinghamshire, UK).

#### 3.8.3. Carrageenan-Induced Paw Edema

To determine the *in vivo* inflammation inhibitory of the *T*. *pubescens* fruiting body extracts, carrageenan-induced rat hind paw edema assay was performed [57]. Six-week-old rats were divided into five groups (each group consisted of five rats). Each group of rats was administered 50 μL of saline mixed with various concentrations of methanol extract (5, 15, and 50 mg/kg per body weight). Then, 30 min after the administration of the saline and the methanol extracts, 0.1 mL of 1% carrageenan was administered onto the subcutaneous surface of the right hind paw. The paw volumes were measured just before carrageenan injection (time 0), and 2, 4, and 6 h after carrageenan injection with a pletismometer (MK-101P, Tokyo, Japan). The increase in paw volume was measured as the difference in paw volume at “0 h” and paw volume at respective hours. The inhibition of inflammation was assessed by the increase of paw volumes, calculated using the following formula.

% increase of paw volume (%I) = [(V_t_ − V_o_)/V_o_] × 100 (6)

where V_t_ represents the final paw volume and V_o_ represents the initial volume of each rat.

### 3.9. Statistical Analysis

All data were expressed as mean ± standard deviations (SD) and SPSS V.13 (SPSS Inc., Chiago, IL, USA) was used for statistical analysis. One-way analysis of variance followed by Tukey multiple comparisons were used to compare means between groups. Differences between means at the 5% (*p* ≤ 0.05) level were considered statistically significant.

## 4. Conclusions

The antioxidant, anti-diabetes, anti-dementia, and inflammation inhibitory activities of the methanol and hot water extracts of *T*. *pubescens* fruiting bodies were studied, and 11 phenolic compounds were discovered in the extracts. The *in vitro* antioxidant, anti-diabetes, anti-dementia, and anti-inflammatory effects of the mushroom were verified by inhibitory activity on α-amylase, α-glucosidase, acetyl- and butyryl-cholinesterase, the production of NO, the expression of iNOS, and *in vivo* carrageenan-induced rat paw edema. Therefore, it is concluded that *T*. *pubescens* fruiting bodies possess good natural antioxidant, anti-diabetes, anti-dementia, and inflammation inhibitory sources.
